# API-2-Induced Cell Migration Is Overcome by Small Molecular Approaches Inhibiting β-Catenin

**DOI:** 10.3390/cimb44120409

**Published:** 2022-11-29

**Authors:** Yonghyo Kim, Myoung-Hee Kang, Yong-Hee Cho

**Affiliations:** 1 Data Convergence Drug Research Center, Therapeutics & Biotechnology Division, Korea Research Institute of Chemical Technology (KRICT), Daejeon 34114, Republic of Korea; 2 Department of Plastic and Reconstructive Surgery, Seoul National University Boramae Medical Center, Seoul 07061, Republic of Korea

**Keywords:** colorectal cancer, cell migration, EMT, resistance to AKT inhibitor, Wnt/β-catenin pathway, PI3K-AKT pathway, FOXO3a

## Abstract

Frequent mutation of *APC* (90%) in advanced colorectal cancer (CRC) results in the simultaneous activation of Wnt/β-catenin and AKT signaling pathways, and the current therapeutic limitations of the AKT inhibitors for treating CRC patients are nuclear β-catenin-induced EMT and bypassing apoptosis. In this study, we discover that the combinatorial treatment of an AKT inhibitor and KY1022, a β-catenin destabilizer, effectively overcomes the current limitations of API-2, an AKT inhibitor, by reducing nuclear β-catenin. Taken together, we demonstrate that the simultaneous suppression of Wnt/β-catenin with the AKT signaling pathways is an ideal strategy for suppressing the AKT-inhibitor-mediated metastasis and for maximizing the therapeutic effects of AKT inhibitors.

## 1. Introduction

Colorectal cancer (CRC) is one of the most deadly cancers in the world [1]. Although recently developed, anti-cancer agents have improved the survival rates of CRC patients by up to 64%, patients with metastatic CRC have only a 12% 5-year survival rate [2], making CRC an extremely lethal disease [2,3,4]. The Wnt/β-catenin and AKT pathways are abnormally activated, mainly due to the *adenomatous polyposis coli* (*APC*) mutation (90%) and *KRAS* mutations (50%), and cooperatively promote the metastatic CRC development [5,6,7,8,9]. Therefore, understanding the molecular mechanisms of their interaction on the metastasis is needed.

The phosphoinositide 3-kinase (PI3K)-V-AKT murine thymoma viral oncogene homolog (AKT) pathway is a major signaling axis that orchestrates the tumor progression and cell migration [10,11]. Activation of AKT disturbs the balance of the cell survival and inhibition of a pro-apoptotic transcription factor of Forkhead box O3 (FOXO3a) [12,13]. FOXO3a is responsible as a controller of cellular processes and a tumor suppressor in the normal state, due to its potent activity for apoptosis and cell cycle arrest [14,15]. As one of the pivotal transcription factors, it presents the central action downstream of the PI3K-AKT signaling, which is negatively regulated by FOXO3a. AKT leads to the phosphorylation of FOXO3a as an inactivated form, translocating it from the nucleus to the cytoplasm and leading to a decrease in its transcriptional activity [16]. In contrast, an inhibited effect of AKT, due to its inhibitors, such as API-2, reverses the release and induces the translocation of FOXO3a from the cytoplasm to the nucleus, inducing the consequent inhibition of its transcriptional activity on the nucleus.

Crosstalk between PI3K-AKT-FOXO3a and Wnt/β-catenin pathways has been discovered to have tumorigenic roles in CRC by causing an interaction between FOXO3a and β-catenin [17]. Synergistic crosstalk between these two transcription factors shows significant resistant effects on the FOXO-induced apoptosis. Especially after the treatment of AKT inhibitors on patient-derived CRC cells, the concomitant highly co-localized expression of FOXO3a and β-catenin on the nuclear region, can be observed [18]. Using these novel clinical implications of dual activation of FOXO3a and β-catenin, the concurrent inhibition of both AKT and the Wnt/β-catenin pathway inhibitors suggests inducing apoptosis in cases of CRC patients’ poor clinical responses. Moreover, these emerging insights suggest that ideal approaches for treating CRC directly correlate with lowering the level of nuclear β-catenin after the treatments of the AKT inhibitors [19].

Given that anti-EMT (epithelial–mesenchymal transition) effects of KY1022, a β-catenin destabilizer, were reported in our previous study [20,21], we aimed to test whether KY1022 suppresses the AKT-inhibitor-mediated cell migration in CRC cells. In this study, we observed that the KY1022 treatment effectively suppresses API-2-induced cell-migration ability via the destabilization of β-catenin, co-accumulated with FOXO3 in the nucleus of the CRC cells harboring *APC* mutation. To understand the inhibition mechanisms of KY1022 involved in the AKT-inhibitor-induced cell migration, we performed immunocytochemistry (ICC) with KY1022, with or without API-2, and we observed that KY1022 effectively decreased the level of β-catenin and FOXO3a in the nucleus of LoVo cells. Next, we aimed to test whether the destabilization of β-catenin via the KY1022 treatment effectively overcomes the API-2-mediated apoptosis resistance. Using flow cytometry analysis, we observed that KY1022 treatment induces the apoptosis of CRC. Given the inhibition results of KY1022 on API-2-induced cell motility and overcoming the effects of KY1022 on API-2-induced resistance to apoptosis, our study suggests that the combination treatment targeting the Wnt/β-catenin pathway with the AKT inhibitors is a promising therapeutic approach to improve the clinical efficiency of treating CRC patients harboring an *APC* mutation.

## 2. Materials and Methods

### 2.1. Cell Culture and Drug Treatment

LoVo cells were provided by Prof. Kang-Yell Choi (Department of Biotechnology, Yonsei University). LoVo cells were tested for authentication using short tandem repeat profiling (Cosmogenetech). LoVo cells were cultured in Dulbecco’s Modified Eagle Medium (DMEM; Gibco, Carlsbad, CA, USA) supplemented with 10% fetal bovine serum (FBS; Gibco). KY1022 was provided by Prof. Gyoonhee Han (Department of Biotechnology, Yonsei University). KY1022 and API-2 (Sigma-Aldrich, St Louis, MO, USA) were dissolved in dimethyl sulfoxide (DMSO; Sigma-Aldrich, St Louis, MO, USA) for the in vitro studies.

### 2.2. Gene-Expression Data Analysis

The gene-expression data analyzed from the NCBI GEO databases are publicly available (accession numbers GSE17536). The data were obtained and processed using Biometric Research Branch (BRB)-Array Tools (National Cancer Institute, Rockville Pike, Bethesda, MD, USA) (version 4.5.0) [22].

### 2.3. Immunocytochemistry and Phalloidin Staining

LoVo cells were seeded to collagen-coated coverslips (500 μg/mL). The cells were treated with DMSO, API-2 (10 μM), KY1022 (20 μM), and the combination treatment with EGF (20 ng/mL) for 18 h. Fixation was performed with the coverslips in 10% neutral formaldehyde for 2 h, followed by the permeabilization with 0.1% Triton X-100 (Sigma-Aldrich, St Louis, MO, USA) for 30 min and blocking in 5% bovine serum albumin (BSA) for 1 h. The coverslips were incubated overnight at 4 °C with anti-β-catenin (BD bioscience, San Jose, CA, USA; #610154) and anti-FOXO3a (Cell Signaling Technology, Danvers, MA, USA; #2497), followed by anti-mouse Alexa Flour 488 (Invitrogen, Waltham, MA, USA; #A11008) or the anti-rabbit Alex Fluor 555 (Invitrogen, #A21428) secondary antibody for 1 h at room temperature. Primary and secondary antibodies were diluted with phosphate-buffered saline (PBS) containing 1% BSA and 1% normal goat serum (NGS; Vector Laboratories, Burlingame, CA, USA). The coverslips were counterstained with 4’, 6’-diamidino-2-phenylindole (DAPI; Sigma-Aldrich) and covered with Gel/Mount media (Biomeda Corporation, Foster City, CA, USA). For staining the cytoskeleton, fixation was performed with 10% neutral formaldehyde, and then the cells were permeabilized, blocked, and incubated with Alexa Fluor 568 phalloidin (Invitrogen, #A12380) for 30 min. Immunofluorescent images were captured using confocal microscopy (LSM 700, Carl Zeiss, Jena, Germany). At least three fields per section were analyzed by Zen V3.1 (Carl Zeiss, Jena, Germany) software (*n* = 4).

### 2.4. Wound-Healing Assay

LoVo cells were seeded in collagen (500 μg/mL)-coated 6-well plates. Once they were grown confluently (80%), the cells were scratched with a 200 μL tip. Then, DMEM (10% FBS) containing EGF (20 ng/mL) was changed with DMSO, API-2 (10 μM), KY1022 (20 μM), and the combination treatment with API-2 (10 μM) and KY1022 (20 μM) for 18 h. Following the treatments, the cells were stained with crystal violet (Sigma-Aldrich). The wound areas were captured and recorded using a microscope (Eclipse Ti, Nikon, Kanagawa, Japan) in a humidified 5% CO_2_ incubator at 37 °C. The number of cells migrated to heal the wound area was quantified using NIS-Elements AR 3.1 software (Nikon). Mean ± s.d. were analyzed, based on three or five biological replicates.

### 2.5. Flow Cytometry Analysis

LoVo cells were seeded (2 × 10^4^ cells) into 6-well plates and incubated with a treatment of DMSO, API-2 (10 μM), KY1022 (20 μM), and a combination treatment with API-2 (10 μM) and KY1022 (20 μM) for 36 h. The cells were collected and resuspended with annexin ν binding buffer containing 10 mM HEPES, 140 mM NaCl, and 2.5 mM CaCl_2_ at 1 × 10^6^ cells/mL. The cells (200 μL) in annexin ν binding buffer were suspended with 5 μL annexin ν -FITC (Sigma-Aldrich) and 10 μL propidium iodide (PI; Sigma-Aldrich) for 15 min in the dark. A total of 800 μL of annexin ν binding buffer was aliquoted to each tube and analyzed by a BD AccuriTM flow cytometer (BD bioscience). Mean ± s.d. were reported, based on three biological replicates.

### 2.6. Statistical Analyses

All statistical analyses were performed using GraphPad Prism 5 Software as described previously [21]. Group differences were analyzed with the Student *t* test. All data were expressed as the means ± s.d. of at least three independent experiments. All statistical tests were two-sided, and *p* values less than 0.05 were considered statistically significant. Significance was denoted as *n.s.* (not significant), * *p* < 0.05, ** *p* < 0.01, and *** *p* < 0.001.

## 3. Results

### 3.1. High Expression of CTNNB1 and FOXO3a Is Associated with Poor Survival in CRC Patients

**Figure 1:**
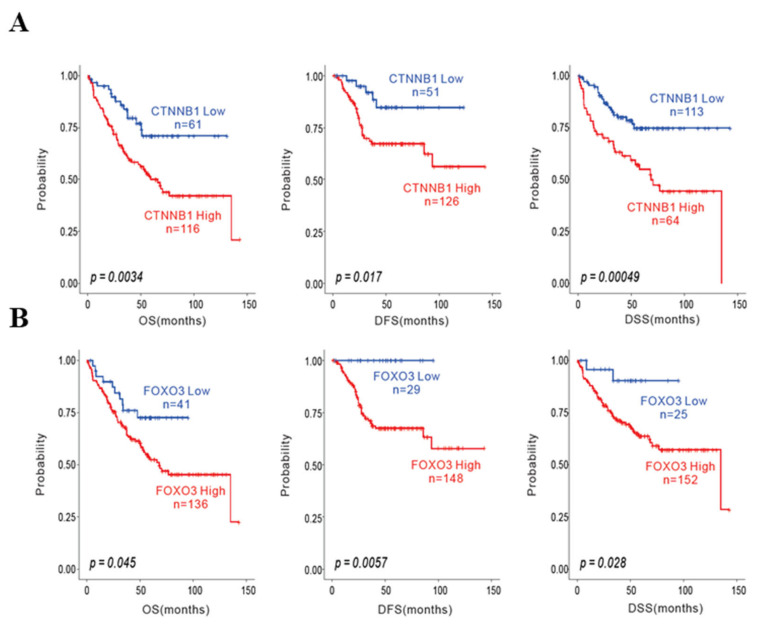
Prognostic values (survival analysis) of the gene expressions in CRC patients, based on the GSE dataset (GSE17536). The effects of the high or low expression of (**A**) CTNNB1 and (**B**) FOXO3a with overall survival (OS), disease-free survival (DFSS), and disease-specific survival (DSS) in CRC patients. OS, overall survival; DFS, disease-free survival; DSS, disease-specific survival.

Accumulating evidence has suggested that the protein levels of β-catenin and FOXO3a are critical factors for interconnecting their signaling crosstalk, promoting the tumorigenesis of CRC [23,24]. We first aimed to confirm the clinical relevance of β-catenin and FOXO3a by analyzing the overall survival (OS), disease-free survival (DFS), and disease-specific survival (DSS) of CRC patients (*n* = 177). We observed that the high expression of CTNNB1 and FOXO3a was associated with a shorter survival in OS, DFS, and DSS in CRC patients (Figure 1). Indeed, CRC patients with a high expression of CTNNB1 also showed a lower survival rate than CRC patients with a low expression of CTNNB1 (Figure 1A). Unexpectedly, CRC patients with a high expression of FOXO3a, known as an apoptosis-inducing gene, showed low OS, DFS, and DSS (Figure 1B), similar to oncogenes, such as CTNNB1, suggesting that FOXO3a has a biological role other than the apoptosis-inducing effects in CRC patients. Taken together, FOXO3a and CTNNB1 play critical roles in promoting the tumorigenesis of CRC.

### 3.2. KY1022 Effectively Destabilizes β-Catenin in the Presence of API-2

**Figure 2:**
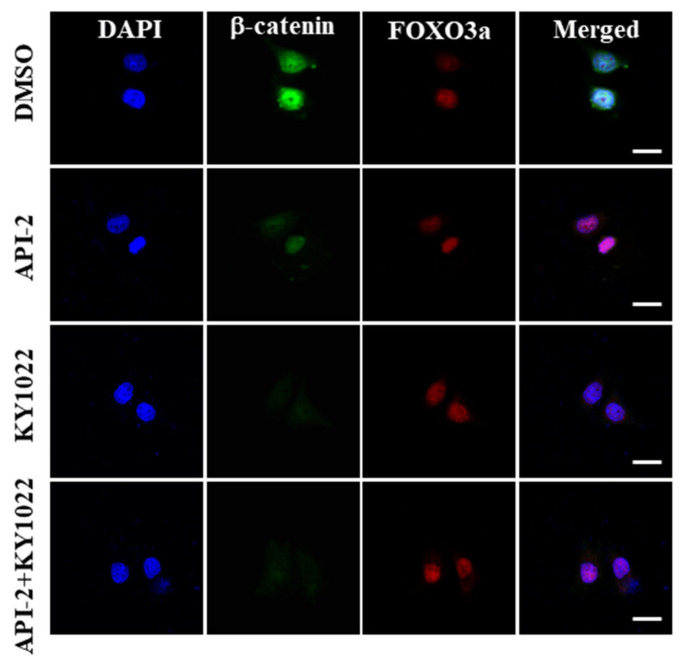
KY1022 destabilizes the nuclear β-catenin in the presence of API-2. The nuclear accumulation of β-catenin (green) and FOXO3a (red) were evaluated by immunocytochemical staining in LoVo cells, with treatment of API-2 (10 μM), KY1022 (20 μM), and the combination treatment. Nuclei were counterstained with DAPI. Representative images were captured using an LSM700 (Zeiss) confocal microscope (*n* = 4 per group). Scale bars = 20 μm.

Given that the translocation of FOXO3a into the nucleus by AKT inhibitors is a current limitation for treating CRC patients with a high expression of nucleus β-catenin [17,19], we aimed to test whether KY1022, a small molecule inhibiting EMT of CRC cells via the destabilization of β-catenin, overcomes the API-2-induced cell migration ability of CRC cells. First, we aimed to test the effects of KY1022 on nuclear β-catenin in the presence of API-2, an AKT inhibitor, using immuno-cytochemical analyses. While API-2 slightly decreases the level of nuclear β-catenin, KY1022 effectively decreases the nuclear β-catenin with or without API-2 treatment. However, the total level of FOXO3a was not changed after KY1022, API-2, and the combination treatment, respectively (Figure 2). The nuclear co-localization of β-catenin and FOXO3a was not shown in the combinatorial treatment of KY1022 with API-2. Overall, the KY1022 treatment effectively destabilizes the nuclear β-catenin, which is the molecular rationale for the API-2-induced cell migration.

### 3.3. KY1022 Reverts the AKT-Inhibitor-Induced Cell Motility and Suppresses the Actin Rearrangement of CRC Cells

**Figure 3:**
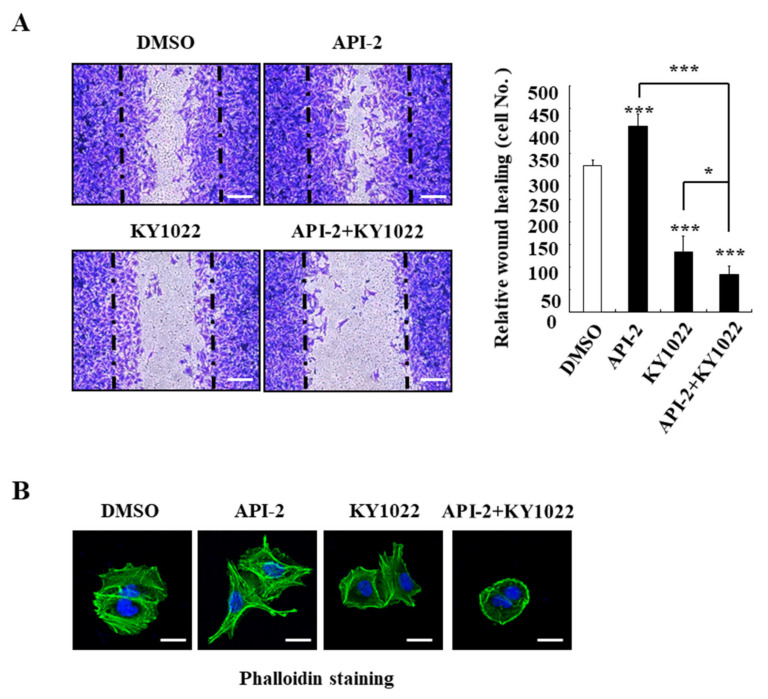
Effects of KY1022 on the API-2-induced cell migration and the actin rearrangement of CRC cells. (**A**) Wound-healing assay using LoVo cells after treatment of API-2 (10 μM), KY1022 (20 μM), and their combinations stained by crystal violet. Representative images (*n* = 4 per group) were visualized by Nikon TE2000U (Kanagawa, Japan) (left panel), and the total number of migrated cells was quantified using NIS-Elements AR 3.1 software (right panel). Scale bar = 100 μm. * *p* < 0.05 and *** *p* < 0.001. (**B**) LoVo cells were incubated with treatment of API-2 (10 μM), KY1022 (20 μM), and their combinations, and the cells were stained with phalloidin (green) and counterstained with DAPI (blue). Representative images were captured by using an LSM700 (Zeiss) confocal microscope (*n* = 4 per group). Scale bars = 20 μm.

Given that the nuclear accumulation of FOXO3a induced by the API-2 treatment induces the cell migration of CRC cells containing high nuclear β-catenin [17,19], we evaluated the inhibition effects of KY1022 on the cell migration using a wound-healing assay and investigated the actin rearrangement of the migratory LoVo cells derived from metastatic tumor nodules. Consistent with recent studies [17,19], API-2 treatment promoted the migration of LoVo cells (Figure 3A). In contrast, the KY1022 treatment suppressed the API-2-induced cell migration ability of the LoVo cells. Next, we aimed to test the combinatorial treatment of KY1022 with API-2 on the actin rearrangement, a prior process of cell migration. Consistent with the migration assay, the actin rearrangement also occurred in the API-1-treated LoVo cells, while the combinatorial treatment with API-2 and KY1022 effectively inhibited the actin rearrangement of the LoVo cells (Figure 3B). On the border of lamellipodia with the structure of filopodia, stressed fibers were formed and enriched in the migratory LoVo cells of the DMSO-treated control. Especially, the API-2 treatment induced cell migration with stressed fibers in the LoVo cells. In contrast, on the peri-nucleus and cytoplasm, KY1022 inhibited actin rearrangement and showed the concentrated actin bundles. The combination treatment with KY1022 and API-2 reduced the stressed fibers and filopodia formation at the cell periphery more than KY1022 alone. In summary, the combination treatment significantly reverts the AKT-inhibitor-induced cell motility and suppresses the actin rearrangement of CRC cells.

### 3.4. KY1022 Overcomes the AKT-Inhibitor-Induced Resistance of Apoptosis in CRC Cells

**Figure 4:**
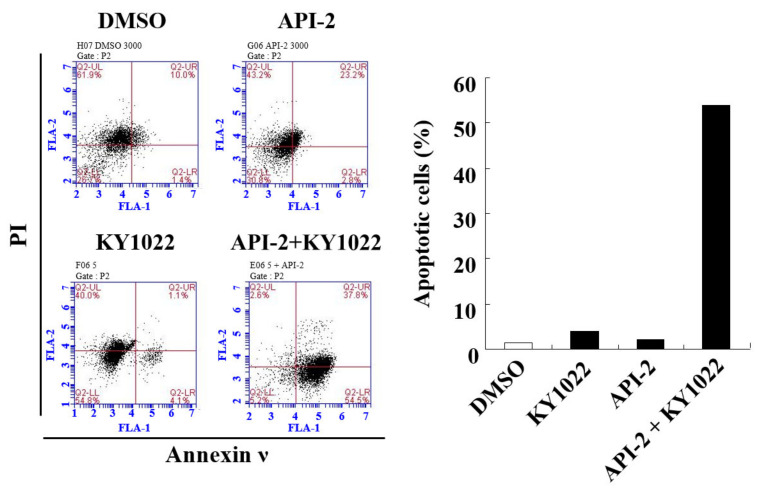
Effects of KY1022 on the API-2-induced resistance of the apoptosis in CRC cells. For each treatment of DMSO, API-2 (10 μM), KY1022 (20 μM), and the combination treatment, the flow cytometry analyses of the LoVo cells were stained with PI and annexin ν using a BD Accuri^TM^ flow cytometer (left). Percentage of the early apoptotic cells expressing annexin ν and not expressing PI were quantified (right).

The wnt/β-catenin pathway plays a major role in the apoptosis of CRC cells [25]. To determine the involvement of the Wnt/β-catenin pathway in apoptosis, we tested the anti-apoptotic activity of API-2 and KY1022 using flow cytometry analysis in LoVo cells. The combination treatment with API-2 and KY1022 significantly increased the number of early apoptotic cells expressing annexin ν without PI than every single treatment, respectively (Figure 4). These results revealed that the combination treatment with API-2 and KY1022 overcomes the API-2-induced resistance to apoptosis in CRC cells.

## 4. Discussion

Through several clinical trials worldwide, various inhibitors targeting the PI3K-AKT signaling pathways have been investigated to evaluate their promising efficacies on cancers [26]. Although significant efforts are ongoing to develop promising drugs targeting PI3K and AKT, most CRC patients have failed to show results due to the limited efficacy and poor clinical responses. The complexity of the PI3K-AKT signaling pathways exhibits extensive interconnections of various crosstalk nodes with other pathways, resulting in intrinsic and acquired resistances [27].

The significant interconnection with the PI3K-AKT pathways and the Wnt/β-catenin pathways has a critical role in regulating cellular growth, homeostasis, and maintenance via modulating the intestinal stem cells and their differentiation [5,6]. However, the aberrant activation of these pathways is well-known as a cooperative cause of tumorigenesis via various mutations [7]. In CRC, the *APC* mutation occurring in 90% of CRC patients is well characterized as an essential factor for the initiation of CRC [28,29]. This mutation is observed as a risk factor for CRC patients treated with AKT inhibitors, implying that the oncogenic activation of the Wnt/β-catenin pathway correlates with resistance mechanisms for inhibitors targeting the PI3K-AKT pathways [19]. In accordance, highlighting the interconnected mechanisms and elucidating the therapeutic targets in CRC could be essential steps to understanding the drug resistance related to the PI3K-AKT pathways.

In recent research, with the overexpression of the Wnt/β-catenin pathway inducing tumor progression in CRC, a key transcription factor, FOXO3a has been reported as an accelerator overexpressing its target genes through binding with β-catenin. In consequence, highly nuclear β-catenin promotes the cell migration and confers resistance on the FOXO3a-mediated apoptosis by treating the AKT inhibitors in CRC cell lines [17]. Indeed, additional reports, including clinical data, demonstrate that the high accumulation of nuclear β-catenin could be a reliable predictive biomarker for evaluating the resistance to AKT inhibitors in CRC patients [19]. Further, clinical evidence from AKT-inhibitor-treated CRC patients shows that high amounts of nuclear β-catenin content induce a shorter progression-free survival (PFS) in differential clinical trials [19]. Therefore, to overcome the AKT-inhibitor-induced resistance to apoptosis in CRC, we aimed to evaluate the therapeutic effect of a decrease in nuclear β-catenin via the KY1022 treatment.

Metastasis is a major cause of cancer-related death and consists of a sequence of processes, including EMT, intravasation, and colonization of secondary organs [30,31]. Among metastasis processes, we tested whether KY1022, an inhibitor of the Wnt/β-catenin pathways, attenuated the API-2-induced cell migration, which is an initial step of cancer metastasis. We observed that KY1022 suppresses the API-2-induced cell migration ability via the destabilization of β-catenin co-accumulated with FOXO3a in the nucleus of LoVo cells harboring *APC* mutations. The combination treatment with KY1022 and API-2 reverted the AKT-inhibitor-induced cell motility and suppressed the actin rearrangement of LoVo cells. Although this study only focused on cell migration, an initial step of multiple metastasis processes, the results clearly showed that the strategies suppressing the Wnt/β-catenin pathways effectively attenuate the API-2 mediated-cell migration. Based on this study, further validation of KY1022 in API-2-induced-other metastasis processes, followed by cell migration, would be helpful for the KY1022 combinatorial treatment with API-2. In addition, resistance to apoptosis via the API-2 treatment is also a problem. The combination treatment overcame the API-2-induced apoptotic resistance in LoVo cells, providing a beneficial option to treat metastatic CRC patients. Taken together, our results on the combination treatment, which showed synergistic effects between KY1022 and an AKT inhibitor, demonstrate a significantly higher clinical efficacy than either agent alone in metastatic CRC patients, accompanied with improvements for a worse/poorer prognosis.

## Data Availability

Not applicable.
